# Contemporary Strategies in SmI_2_ Catalysis: A Reagent Reborn

**DOI:** 10.1002/anie.202519678

**Published:** 2025-10-13

**Authors:** Jack I. Mansell, Ciro Romano, David J. Procter

**Affiliations:** ^1^ Department of Chemistry University of Manchester Oxford Road Manchester M13 9PL UK

**Keywords:** Catalysis, Electrochemistry, Photochemistry, Radicals, Samarium

## Abstract

The strategies adopted in the development of Sm^II^‐catalyzed processes are critically evaluated. In particular, we examine promising approaches using stoichiometric sacrificial reductants, electrochemical methods, photochemical processes, and electron recycling through back electron transfer to Sm. While each strategy has considerable potential for facilitating catalytic Sm^II^ transformations, they also face specific challenges relating to efficiency, selectivity, scalability, and generality. We highlight the strengths and limitations of each approach, discuss recent advances, and identify challenges that remain. By offering an in‐depth analysis of the current state‐of‐the‐art in Sm^II^ catalysis, we aim to guide future research to complete the reinvention of SmI_2_ as a tool for sustainable catalysis.

## Graphical abstract

The rich chemistry of the single electron transfer reagent SmI_2_ has been widely exploited in synthesis. Recent years have seen significant progress in SmI_2_‐catalysis, with provision of a suite of systems that may evolve to provide the efficiency, practicality, and generality needed for widespread adoption. This minireview provides a critical evaluation of strategies for Sm^II^‐catalysis and maps out a course that will complete the reinvention of SmI_2_ as a tool for sustainable catalysis.

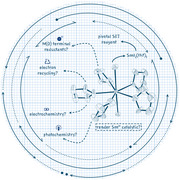

## 1 Introduction

**Scheme 1:**
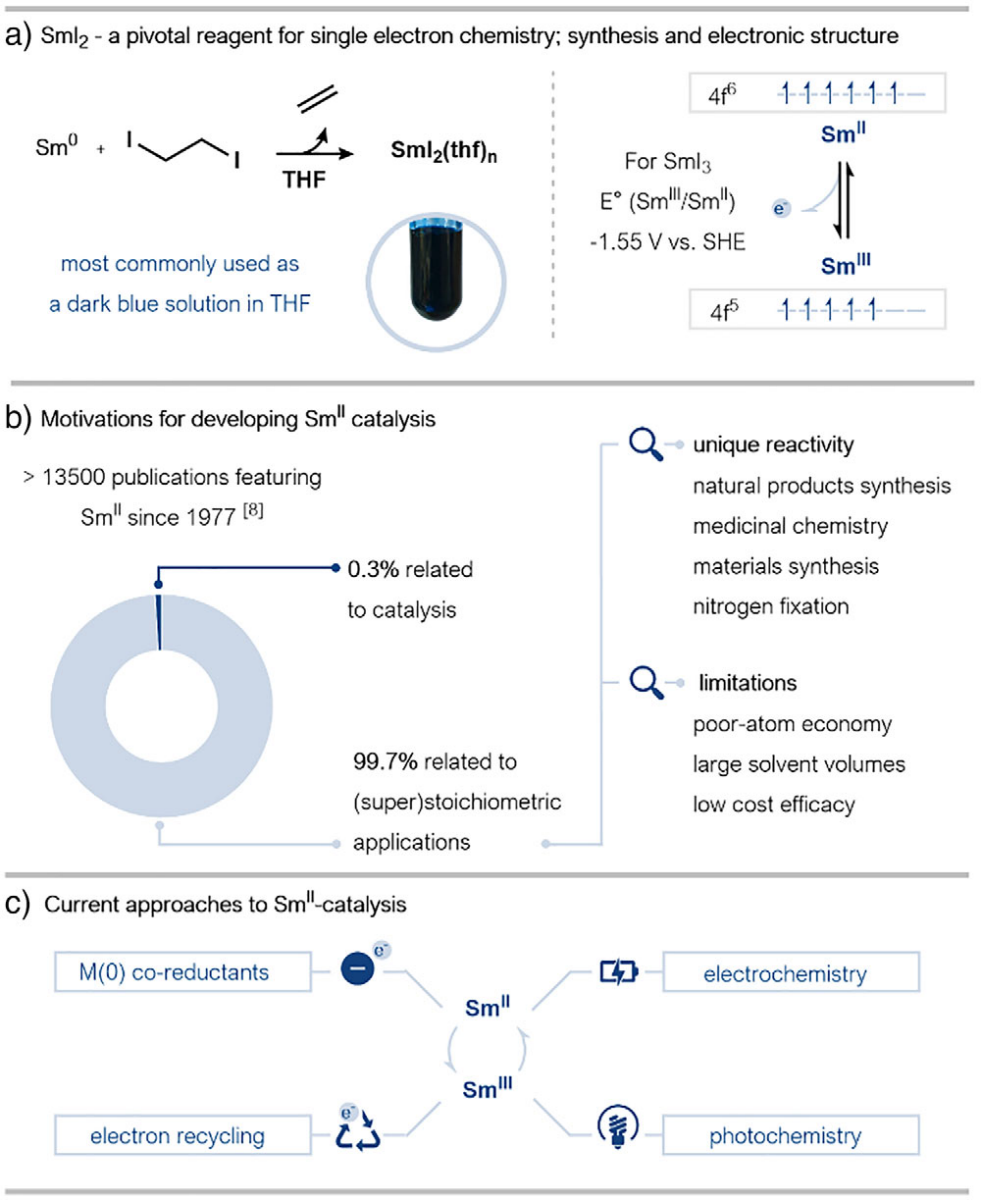
a) The most common method for the preparation of SmI_2_ as a solution in THF and the reagent's electronic structure. The photograph of SmI_2_ as a solution in THF was taken by the authors. b) The importance of SmI_2_ chemistry across research areas and the scarcity of catalytic studies in the context of its widespread use. c) Current strategies employed in SmI_2_ catalysis. THF/thf: tetrahydrofuran; SET: single electron transfer; SHE: standard hydrogen electrode.

Driven by the promise of bringing the rich chemistry of samarium(II) iodide (SmI_2_) to the field of catalysis, approaches to Sm^II^ catalysis have received increasing attention in recent years. Since the first user‐friendly preparation of SmI_2_, by Kagan and coworkers in 1977, the divalent lanthanide reagent has emerged as a highly versatile and selective single‐electron transfer (SET) reducing agent, capable of mediating reductions that are challenging to achieve otherwise (Scheme 1a).^[^ ^1^, ^2^ ^]^ As the flagship Sm^II^ reagent, the deployment of SmI_2_ has been documented in many reports, with the vast majority (>99%) featuring its use as a (super)stoichiometric reagent across a broad range of synthetic disciplines (Scheme 1b).^[^ ^3^, ^4^, ^5^, ^6^, ^7^, ^8^, ^9^, ^10^ ^]^ However, the need to use stoichiometric amounts of the reagent is a limitation of SmI_2_‐mediated reactions, resulting in significant volumes of waste and raising concerns around sustainability. While the stoichiometric use of SmI_2_ has been widely adopted in synthesis, the need to develop catalytic versions of its reactions has long been appreciated. The regeneration of Sm^II^ under catalytic conditions presents significant challenges, largely owing to the stability of Sm^III^ and its affinity for reaction products. Regenerating Sm^II^ efficiently, using benign, inexpensive sacrificial reductants and mild conditions, is key to developing catalytic processes that are sustainable and resource‐efficient. The importance of rendering SmI_2_‐mediated reactions catalytic likely extends beyond sustainability; for example, the use of catalytic quantities of SmI_2_ may unlock reaction pathways hidden by the presence of excess reagent. Among the many avenues for application of new catalytic processes, the well‐known suitability of SmI_2_ for the selective construction of intricate molecular architectures will benefit from a transition to a cost‐effective and scalable catalytic regime. This aspect of SmI_2_‐catalysis is particularly relevant in the context of fine chemical synthesis, pharmaceuticals, and materials science, where efficient and selective catalytic cross‐coupling is of paramount importance.

Over the past three decades, a range of approaches have been explored to regenerate active Sm^II^ species from Sm^III^—a process key to catalysis; the use of stoichiometric sacrificial metal(0) reductants, electrochemical methods, electron recycling through back electron transfer (BET) to Sm, and photochemical regeneration represent some of the most promising strategies explored to date. While these methods hold potential for facilitating catalytic cycles, each comes with its own set of challenges—whether related to efficiency, selectivity, scalability, or generality. Electrochemical strategies, for example, are straightforward to run once the initial barrier to implementation is overcome but can suffer from issues of functional group tolerance. Electron recycling, on the other hand, which harnesses the reversible nature of radical formation using SmI_2_, avoids the use of sacrificial reductants but is currently limited to a small subset of reactions.

Rather than a more traditional review,^[^ ^11^, ^12^, ^13^ ^]^ this treatise aims to provide an in‐depth understanding of the state‐of‐the‐art in Sm^II^‐catalysis, critically unpicking strategies by highlighting their strengths and limitations, while examining the overall progress made and the gaps that remain. Furthermore, the review aims to offer insight into the future of the field, focusing on the continuing pursuit of generally applicable and sustainable Sm^II^ catalytic strategies that are compatible with both the extensive, established chemistry of SmI_2_, and powerful new processes that are not possible under stoichiometric regimes.

## 2 Sacrificial M(0) Co‐Reductants

**Scheme 2:**
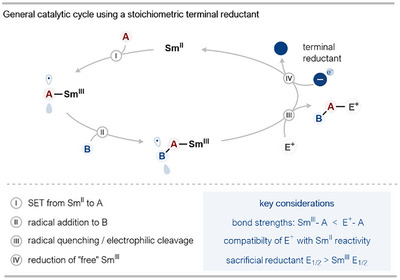
General cycle using a stoichiometric sacrificial terminal reductant for Sm^II^‐catalysis.

To address the limitations of stoichiometric reagent use inherent in early SmI_2_ chemistry, the first strategies pursued the utilization of sacrificial terminal metal reductants. This approach employs stoichiometric amounts of a less expensive, readily available reducing agent, typically a low‐valent metal, to regenerate the active Sm^II^ species from the Sm^III^ byproduct formed after substrate transformation, thereby enabling catalytic turnover. A generalized catalytic cycle capturing this strategy is shown in Scheme 2. Reductive SET from Sm^II^ to a substrate (A) initiates the desired radical chemistry, often involving radical addition or cyclization events. When SET is an inner‐sphere process—for example, carbonyl reduction—resultant radicals are bound to Sm^III^. Radical reactions typically generate product‐bound Sm^III^ intermediates. Subsequent heterolytic cleavage of the product from the Sm center, often facilitated by a stoichiometric electrophilic additive (E⁺), yields “free” Sm^III^ species. These Sm^III^ species are then reduced back to the active Sm^II^ state by an excess of stoichiometric terminal reductant, thus closing the catalytic cycle. Key considerations for the successful implementation of this strategy are a thermodynamic driving force to facilitate cleavage of the Sm^III^–product bond (Sm^III^–A) and formation of the electrophile–product complex (E⁺–A); the chosen electrophilic additive must be inert to reduction by Sm^II^; and, crucially, the sacrificial reductant must possess a reduction potential sufficient to reduce Sm^III^ back to Sm^II^.

### 2.1 Early Approaches to Sm^II^ Regeneration Using Stoichiometric Sacrificial Reductants

**Scheme 3:**
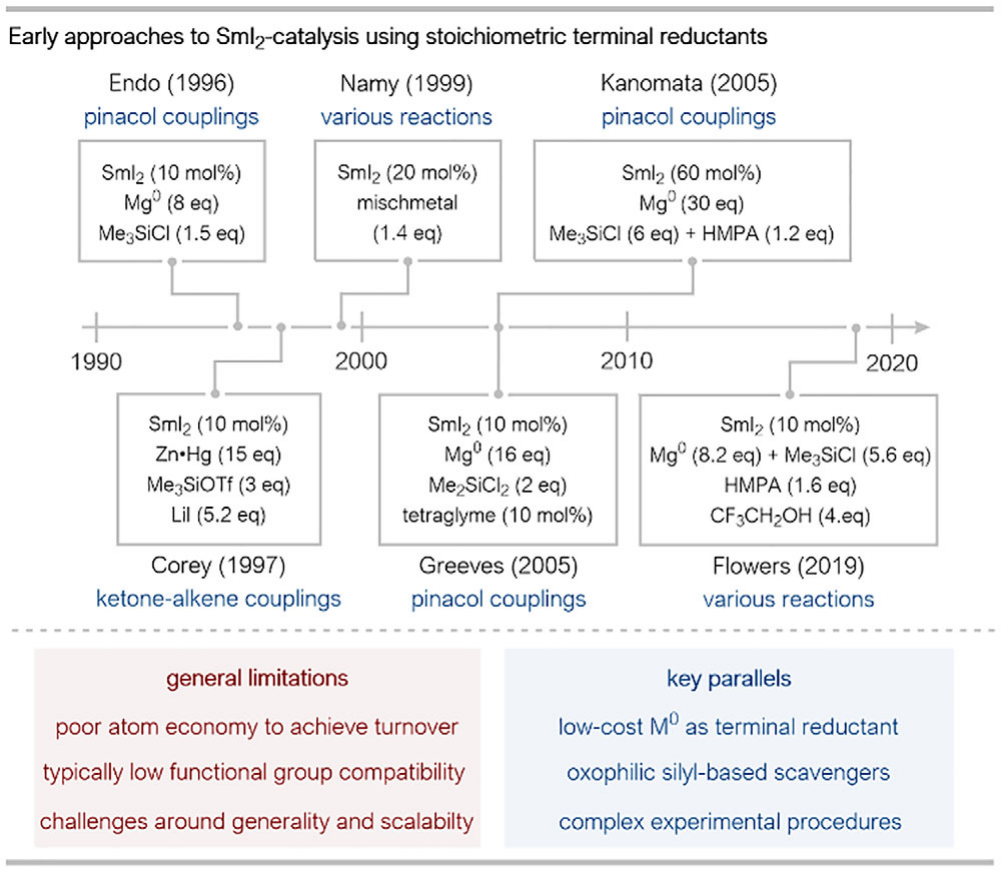
Timeline of the development of protocols for SmI_2_ catalysis using stoichiometric terminal metal reductants. HMPA: hexamethylphosphoramide, tetraglyme: tetraethylene glycol dimethyl ether, Tf: 1,1,1‐trifluoromethanesulfonyl.

Pioneering work demonstrating the feasibility of a catalytic Sm^II^ approach using stoichiometric co‐reductants emerged in the mid‐to‐late 1990s (Scheme 3). In 1996, Endo and coworkers reported the first nonelectrochemical SmI_2_ catalytic system, utilizing Mg(0) as the terminal reductant in conjunction with chlorotrimethylsilane (Me_3_SiCl) as an electrophilic additive, to help convert Sm^III^‐alkoxide intermediates to active Sm^II^ species. Endo's system was used specifically for the homo pinacol couplings of aldehydes and ketones.^[^ ^14^ ^]^ Shortly after, in 1997, Corey and coworkers applied a SmI_2_/Zn(Hg) amalgam system in catalytic ketone annulation reactions that produce spiro‐lactone products.^[^ ^15^ ^]^ In this system, lithium iodide (LiI) and trimethylsilyl triflate (Me_3_SiOTf) were found to be crucial for improving catalytic turnover; analogous to the system of Endo, Me_3_SiOTf acts as an electrophilic additive to trap Sm^III^‐alkoxide intermediates as silyl ethers and liberates “free” Sm^III^.

In 1999, Namy and coworkers established the use of mischmetal, an inexpensive alloy of early lanthanides, as an effective terminal reductant for SmI_2_‐catalysis.^[^ ^16^ ^]^ The authors propose that regeneration of Sm^II^ occurs through reduction of Sm^III^‐alkoxides by mischmetal (or constituent metals like Ce/La); a process that potentially involves salt metathesis steps and organosamarium intermediates. Significantly, this system obviated the need for silyl electrophiles in some coupling reactions, simplifying the protocol but further increasing the amount of rare‐earth metal waste.

Collectively, these seminal studies, falling within a 3‐year period, established the viability of employing sacrificial low‐valent metal reductants to achieve catalytic turnover in SmI_2_ chemistry and represent a critical first step toward more sustainable practices. They also highlight the complexities involved, particularly the crucial role of additives in facilitating the release of Sm^III^ and efficient Sm^II^ regeneration, and set the stage for further method refinements.

Building on the foundational work of Endo, in 2005, Greeves and coworkers employed tetraethylene glycol dimethyl ether (tetraglyme) as a chelating additive in conjunction with SmI_2_, Mg(0), and dichlorodimethylsilane (Me_2_SiCl_2_; found to be superior to Me_3_SiCl for catalysis) in both inter‐ and intramolecular pinacol couplings.^[^ ^17^ ^]^ In the same year, Kanomata and coworkers reported a Mg(0)/HMPA/Me_3_SiCl‐system for SmI_2_‐catalyzed intramolecular pinacol couplings; however, larger excesses of sacrificial reductant and electrophile were required to achieve catalytic turnover.^[^ ^18^ ^]^ More recently, in 2019, Maity and Flowers conducted detailed mechanistic studies on Endo's Mg(0)/Me_3_SiCl‐based catalytic system, finding that the introduction of a proton donor, such as trifluoroethanol, enhanced efficiency.^[^ ^19^ ^]^ Their investigations provided compelling evidence that the active catalyst evolves over the reaction time course: SmI_2_ transforms into the more potent reductant, SmCl_2_, as chloride is generated from the silyl chloride additive. This Sm salt metathesis helps explain the ability of some catalytic systems to reduce substrates normally recalcitrant to stoichiometric SmI_2_.

More recently, the use of stoichiometric metal reductants to regenerate Sm^II^ species has been applied in the context of nitrogen fixation, wherein Sm‐ and Mo‐based catalysts work cooperatively to convert dinitrogen to ammonia.^[^ ^20^, ^21^ ^]^ An alternative Sm^II^ source has also been employed in Sm^II^‐catalyzed pinacol couplings; allylSmBr and carboxylic acids (RCO_2_H) are proposed to form RCO_2_SmBr,^[^ ^22^ ^]^ which can be used with trimethylsilylbromide (Me_3_SiBr) and Mg(0) for catalysis, akin to the seminal report by Endo and coworkers.^[^ ^14^ ^]^

Clear parallels across these studies are the use of an inexpensive, sacrificial low‐valent metal reductant and silylating agents; the latter are almost universally required to cleave the strong Sm^III^─O bonds in products of carbonyl coupling, thereby liberating Sm^III^ for reduction. Additionally, the choice of solvent in almost all cases is tetrahydrofuran (THF), the solvent almost invariably used for the preparation and utilization of Sm^II^ species, due to the stability and solubility of the reagents in THF. The exploration of co‐ligands and/or additives (e.g., LiI, HMPA, glymes, proton donors) to modulate reactivity, selectivity, or solubility constitutes another shared theme between studies, demonstrating the sensitivity of Sm^II^ chemistry to the coordination environment of Sm. Finally, nearly all reports highlight the need for careful optimization of reaction conditions, including reagent stoichiometry, addition rates, and the choice of additives to achieve efficient catalysis. Despite successfully reducing the reliance on stoichiometric SmI_2_, the sacrificial metal reductant strategy faces limitations regarding overall efficiency, cost, and waste generation. While terminal metals like Mg(0) or Zn(0) are cheaper than Sm, their super‐stoichiometric use generates significant metallic salt waste. In addition to being an additional waste stream, these metal salts can also interfere with the reaction, for instance, by sequestering supporting ligands from the Sm‐center and complicating Sm‐speciation. Finally, the essential additives, particularly silylating agents required for product release, are often used in (super)stoichiometric quantities, further reducing the atom economy of the overall processes.

### 2.2 Protonolysis of Sm^III^‐Alkoxides

**Scheme 4:**
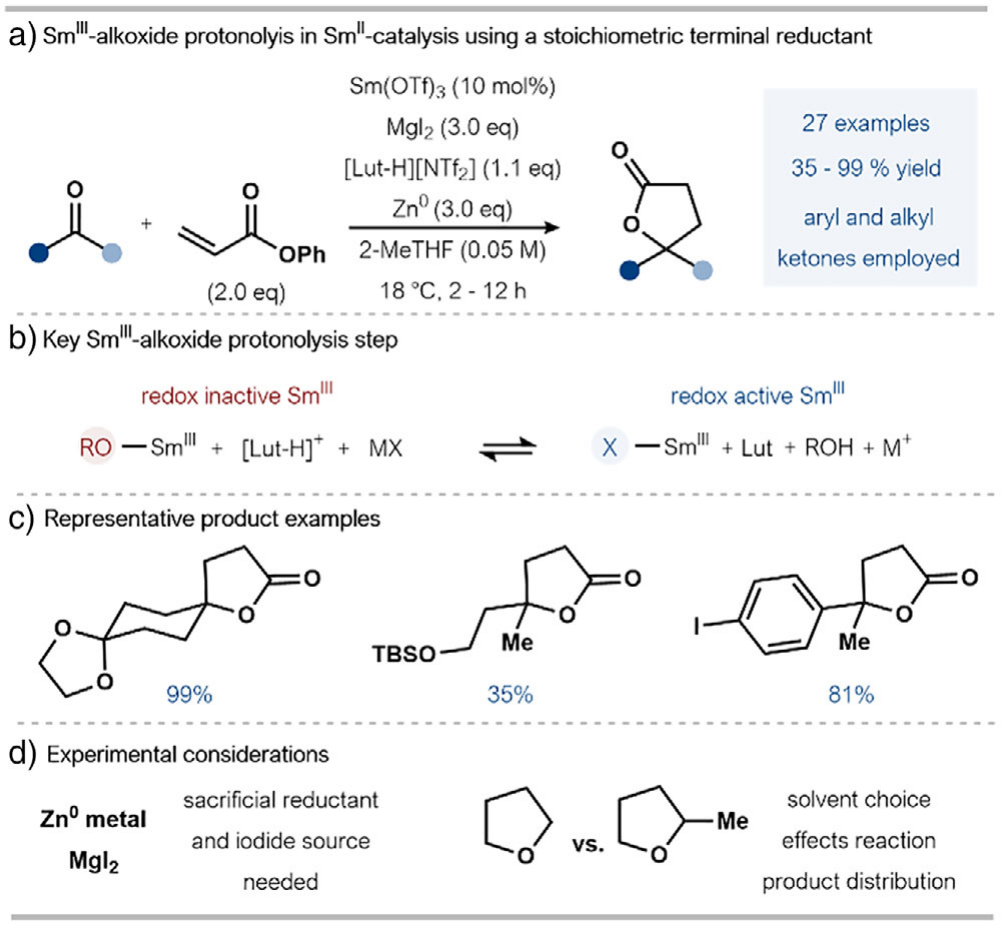
a) Sm^II^‐catalyzed ketone–alkene coupling/cyclization using a Sm^III^‐alkoxide protonolysis approach. b) The generation of a reducible Sm^III^ species is key to the use of a milder stoichiometric reductant and efficient catalysis. c) Selected examples illustrating the reaction scope. d) Key experimental considerations for the protocol. Lut: 2,6‐dimethylpyridine (2,6‐lutidine), TBS: *tert‐*butyldimethylsilyl, Tf: 1,1,1‐trifluoromethanesulfonyl, THF: tetrahydrofuran.

In 2024, Reisman and Peters and their coworkers reported an important addition to the field of Sm^II^‐catalysis using zinc as a stoichiometric co‐reductant (Scheme 4a).^[^ ^23^ ^]^ A central focus of this study was the longstanding challenge to efficient catalysis posed by the strong Sm^III^─O bonds formed during ketyl radical generation from carbonyl substrates and that are present in the products of cross‐coupling.

To address this, the authors utilized a pyridinium salt in combination with a stoichiometric iodide source, MgI_2_, to cleave the Sm^III^─O bond and generate SmI_3_ (Scheme 4b). Pyridinium salts had previously been used by Gansäuer in titanium redox catalysis to protonate Ti^IV^─oxygen bonds had not been applied to Sm^II^‐catalysis.^[^ ^24^, ^25^, ^26^ ^]^ Subsequent SET reduction of SmI_3_ by Zn(0), a milder reductant compared to Mg(0), amalgams, or mischmetal—regenerated Sm^II^ and closed the catalytic cycle. Another striking feature of the approach is the use of an easier‐to‐handle Sm^III^ salt as a precatalyst from which SmI_2_ is formed in situ.

Using the ketone–alkene coupling that Corey employed in his early study on SmI_2_ catalysis,^[^ ^15^ ^]^ Reisman and Peters showed that their catalytic system is impressively broad, accommodating both aliphatic and aromatic ketones, with functional groups such as silyl ethers and aryl halides untouched under the catalytic conditions (Scheme 4c).

**Scheme 5:**
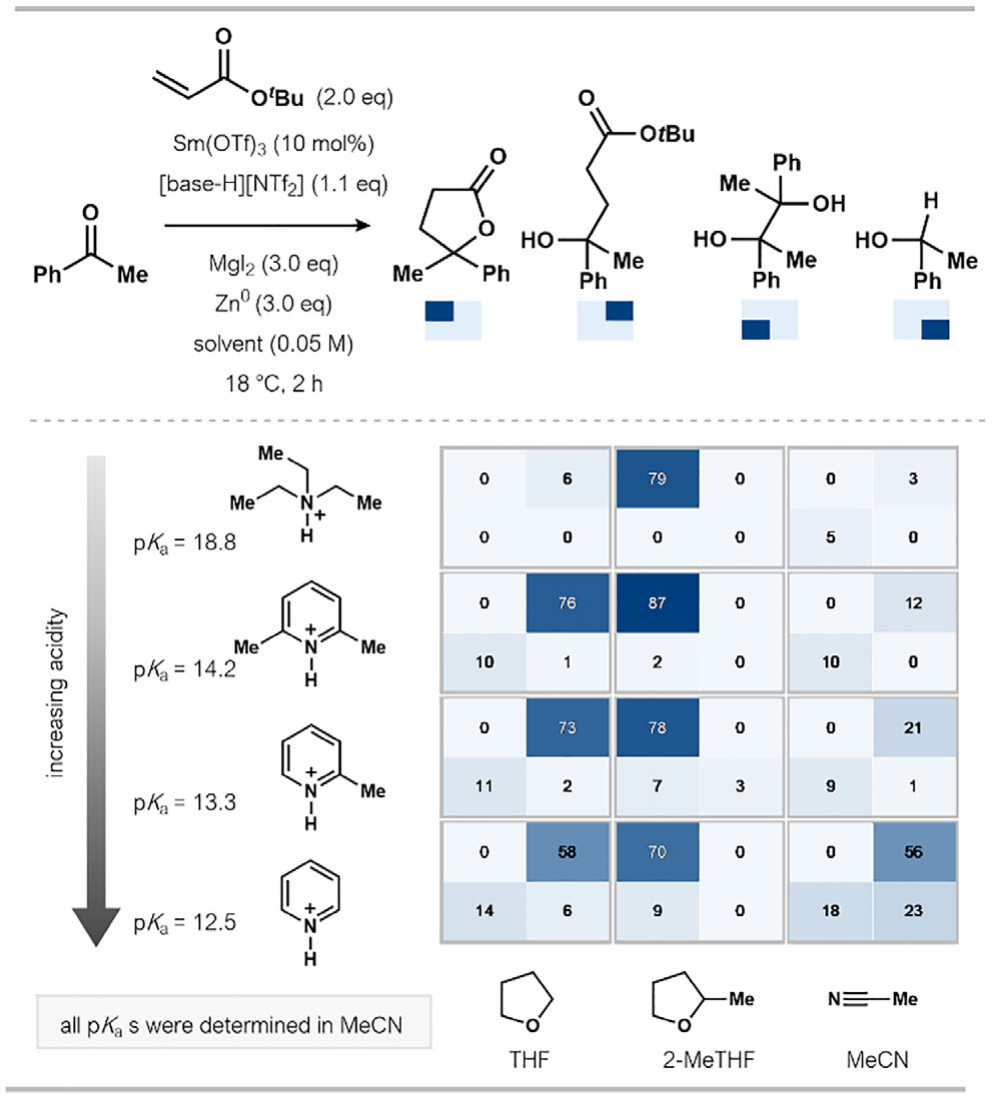
The effect of the proton source and reaction solvent on the chemoselectivity of the Sm^II^‐catalyzed ketone–alkene coupling. ^1^H NMR yields are reported.

As part of the reaction optimization, several pyridinium/ammonium salts were trialed, ranging in relative acidity (p*K*_a_ calculated in MeCN), in combination with different solvents (using *tert*‐butyl acrylate as the coupling partner). The yields obtained with selected combinations are illustrated in the heatmap in Scheme 5.

Solvent choice proved crucial for effective formation of the targeted lactone product; when THF was employed in lieu of 2‐MeTHF, the uncyclized hydroxy ester product accounted for most of the mass balance. Based on these findings, in conjunction with cyclic voltammetry studies, the team suggested that the switch of 2‐MeTHF for THF results in different protonolysis kinetics. Furthermore, choice of solvent is also important as solvent binding to the Sm metal center impacts the ability of the substrate to also coordinate to Sm ahead of inner‐sphere SET. When acetonitrile (MeCN)—a more coordinating solvent—was employed, the homo‐coupled diol was detected; the authors suggest that this is due to faster Sm^III^—O protonolysis outcompeting addition to the acrylate. The authors also noted an increase in yield of the hydroxy ester product with more acidic conditions when MeCN was employed as solvent.

The choice of phenyl acrylate as a coupling partner appears important to achieve high yields of lactone products during studies on the reaction scope; for example, the use of 1,1,1‐trifluoroethyl acrylate results mainly in pinacol product formation, with a low yield of lactone observed.

In summary, this Sm^III^‐alkoxide protonolysis strategy delivers a considerably more attractive platform for catalysis when compared to earlier approaches using stoichiometric metal reductants. However, issues around atom economy remain with the use of stoichiometric Zn(0) and MgI_2_, while the complex interplay of additives and glovebox set‐up add a degree of operational complexity. Future application of this highly promising approach to other SmI_2_‐mediated transformations could lead to a truly general Sm^II^‐catalytic system.

## 3 Electron Recycling

**Scheme 6:**
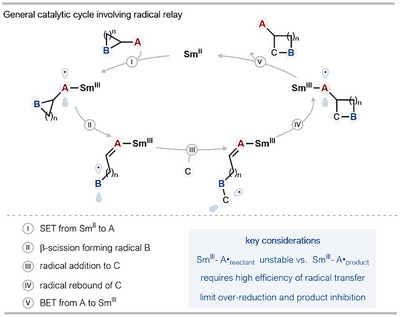
General catalytic cycle for Sm^II^‐catalysis exploiting a radical relay mechanism. SET: single electron transfer, BET: back electron transfer.

Radical relay catalysis, or “electron recycling,” is a contemporary alternative approach to Sm^II^‐catalysis that boasts excellent atom economy.^[^ ^27^ ^]^ Current examples of the approach involve reduction of a functional group adjacent to a strained ring, which undergoes radical fragmentation to promote cycloaddition processes. A generalized catalytic cycle begins with SET reduction of a functional group (A) with Sm^II^ to form a Sm^III^–A bond (Scheme 6). As the newly generated radical is adjacent to a strained‐ring system, the radical can undergo β‐scission to generate a radical at B. Subsequent radical trapping by C, followed by a cyclative radical rebound, generates a radical back at A. Finally, it is thought that back‐electron transfer (BET) to Sm^III^ cleaves the Sm^III^─A bond, liberates product, and regenerates the Sm^II^ catalyst. To date, SmI_2_ radical relay catalysis has been used to form two new C─C bonds in cycloadditions. Key considerations for the successful implementation of this strategy include i) the instability of the initial radical, due to fragmentation of the adjacent strained ring, versus the product radical, and ii) the efficiency of radical transfer throughout the catalytic cycle, versus radical reduction by Sm^II^ to form carbanions (radical‐polar crossover).

### 3.1 Ring‐Opening of Cyclopropyl Ketones

**Scheme 7:**
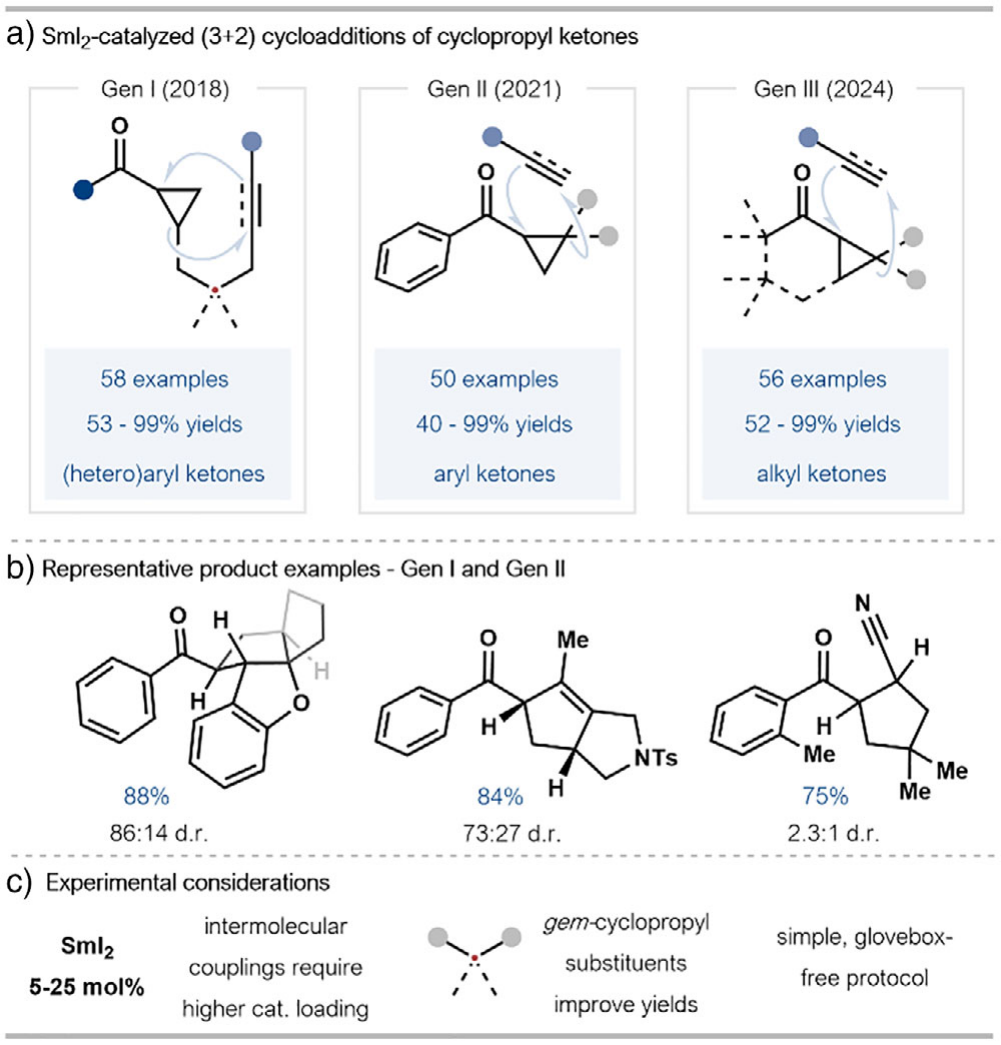
a) Radical relay mechanisms drive SmI_2_‐catalyzed (3 + 2)‐cycloadditions. b) Representative product examples formed by the intra‐ and intermolecular coupling of (hetero)aryl ketones catalyzed by SmI_2_. Reaction conditions: intramolecular couplings, SmI_2_ (20 mol%), THF (0.025 M), 65 °C, 20 min; intermolecular coupling, acrylonitrile (5.0 equiv), SmI_2_ (25 mol%), THF (0.1 M), 55 °C, 45 min. Ts: toluenesulfonyl. c) Highlighted experimental considerations.

Our group has reported a series of studies showcasing the use of radical relay strategies to drive SmI_2_ catalysis. Crucially, this approach does not need stoichiometric co‐reductants and electrophilic additives; in most cases, only catalytic SmI_2_ alone is used. Processes have evolved from intramolecular radical (3+2)‐cycloadditions to intermolecular coupling reactions of aryl cyclopropyl ketones and, more recently, less‐reactive alkyl ketones (Scheme 7a).^[^ ^28^, ^29^, ^30^ ^]^

Earlier processes involving aryl ketone substrates enabled access to complex Csp^3^‐rich polypentacyclic systems (intramolecular couplings) and highly decorated cyclopentyl systems (intermolecular couplings) in high yields and with moderate diastereocontrol (Scheme 7b).^[^ ^28^, ^29^ ^]^ Comparative analysis of these methods highlights that catalyst loadings need to be higher for intermolecular couplings of aryl ketones and high yields require geminal alkyl substituents on the cyclopropyl ring; the radical formed upon β‐scission is stabilized by the alkyl substituents. That said, these protocols involve a simple experimental set‐up and do not require exogenous reactants, resulting in reduced waste generation when compared with stoichiometric sacrificial reductant strategies.

**Scheme 8:**
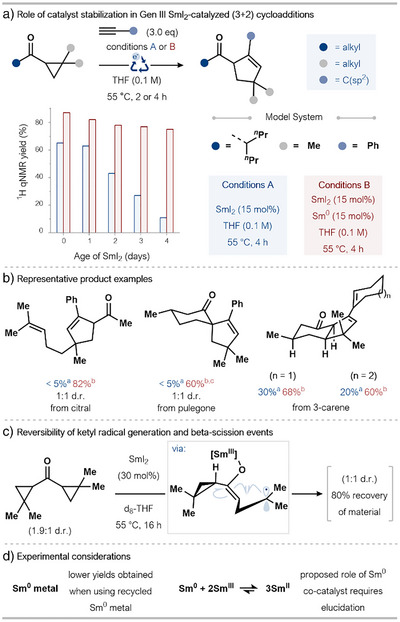
a) SmI_2_ aging studies and the beneficial effect of including substoichiometric Sm(0). b) Representative products from the intermolecular SmI_2_‐catalyzed coupling of alkyl ketones and alkynes/enynes; the positive impact of substoichiometric Sm(0). d.r.: diastereomeric ratio. ^a)1^H qNMR yield using conditions A, ^b)^isolated yield using conditions B, ^c)^30 mol% Sm(0) used. c) Mechanistic experiment supporting the reversibility of both ketone reduction and ring‐opening in SmI_2_‐catalysis. d) Highlighted experimental considerations.

In a recent report, our group has extended the frontiers of the popular field of catalyzed radical (3+2)‐cycloadditions by engaging alkyl cyclopropyl ketones—a challenging substrate class due to their lower redox potentials and low reactivity (Scheme 8a).^[^ ^30^ ^]^

Aging studies on an SmI_2_ solution in THF revealed a gradual decline in catalytic activity over time—likely a consequence of oxidation of Sm^II^ by trace oxygen (Scheme 8a). The addition of Sm(0) effectively mitigated this deactivation, as evidenced by stable yields of product even when older SmI_2_ catalyst was employed. The Sm(0) substoichiometric additive may prevent SmI_2_ deactivation by reducing off‐cycle Sm^III^ species back to Sm^II^, thereby maintaining the activity of the catalyst. Crucially, the introduction of substoichiometric Sm(0) was also found to “switch on” catalysis for the most unreactive substrates (Scheme 8b). In mechanistic studies, it was observed that treatment of a diastereoisomerically enriched *bis*‐cyclopropyl ketone with catalytic SmI_2_ resulted in ablation of the diastereoisomeric enrichment. This suggests that both ketyl radical formation and subsequent β‐scission are reversible (Scheme 8c).

This study provides valuable insight into the challenges around working with substoichiometric amounts of SmI_2_, particularly when reaction rates are low, and offers a way to stabilize the catalyst toward background decomposition. Of the substoichiometric Sm(0) additive used, quantities of the metal can be recovered; however, reduced product yields were obtained when using recycled Sm(0). In‐depth mechanistic studies are needed to ascertain the precise function of Sm(0).

More recently, using the catalytic system and approach of the Procter group, Sharma and coworkers exploited catalytic SmI_2_/Sm^0^ in a cycloaddition reaction for the synthesis of sterically congested boron‐substituted cyclopentenes using *gem*‐diboryl‐substituted cyclopropyl ketone substrates.^[^ ^31^ ^]^

Despite the progress made in developing SmI_2_‐catalyzed cycloadditions of cyclopropyl ketones, the approach has limitations. The need for substoichiometric Sm^0^ when working with more challenging, slow‐reacting substrates complicates reaction setup and introduces an additional layer of handling sensitivity. Moreover, the role of Sm^0^ is not yet fully understood, and this lack of mechanistic understanding may hamper application of the catalytic SmI_2_ approach to other substrate classes.

### 3.2 Ring‐Opening of Other Ketone‐Bearing Strained‐Rings

**Scheme 9:**
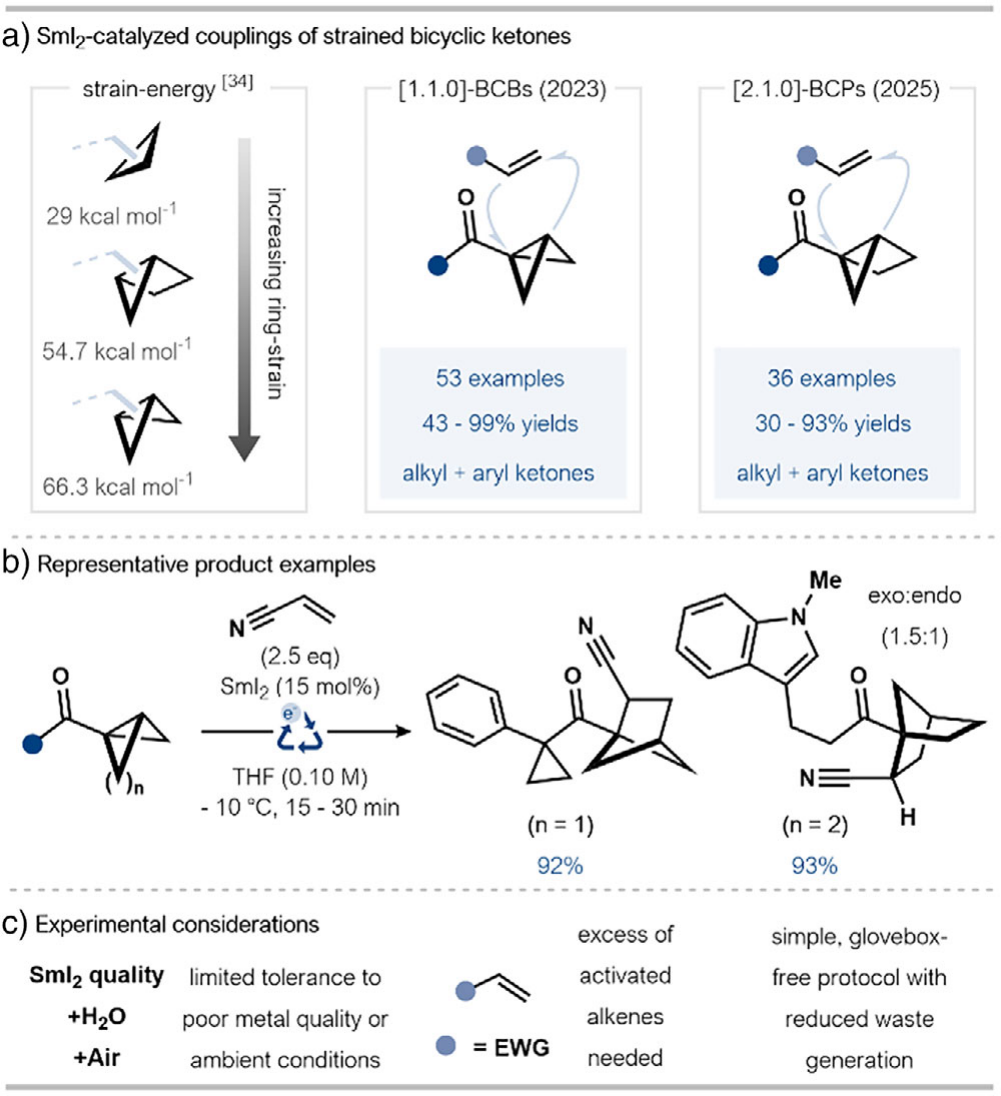
a) Extension of the SmI_2_‐catalyzed cross‐coupling approach to other strained bicyclic ketone systems and alkenes. Relevant experimentally determined strain‐release energies. b) Representative scope examples from the intermolecular coupling of alkyl ketones catalyzed by SmI_2_. c) Highlighted experimental considerations.

Our group has also studied the SmI_2_‐catalyzed fragmentation and coupling of other strained systems bearing exocyclic ketones (Scheme 9a).^[^ ^32^, ^33^, ^34^ ^]^ In 2023, we described a SmI_2_‐catalyzed approach that delivers substituted bicyclo[2.1.1]hexyl (BCH) ketones through the intermolecular coupling of bicyclo[1.1.0]butyl (BCB) ketones and electron‐deficient alkenes, such as acrylonitrile (Scheme 9b).^[^ ^32^ ^]^ These BCH scaffolds are of particular interest as emerging saturated bioisosteres for *ortho*‐ and *meta*‐disubstituted benzenes.^[^ ^35^ ^]^ The process is efficient, operating with low SmI_2_ catalyst loadings—without the need for additives of any kind—and delivering a range of high‐value alkyl and aryl BCH ketones.

Evolution of this SmI_2_‐catalyzed approach allowed the coupling of bicyclo[2.1.0]pentane (housane) ketones with electron‐deficient alkenes to generate functionalized bicyclo[2.2.1]pentane (norbornane) scaffolds; the particular norbornane products are not easily accessible using traditional cycloaddition approaches.^[^ ^33^ ^]^

A comparative analysis of these strain‐release strategies highlights the versatility of the SmI_2_‐catalyzed radical relay approach. While the couplings of BCB ketones could tolerate a catalyst loading as low as 5 mol%, in specific cases, higher loadings of SmI_2_ were needed for housane ketones. However, both systems demonstrate excellent functional group tolerance and broad substrate scope, and reactions are typically complete within a matter of minutes.

A limited tolerance to the presence of H_2_O and O_2_ was observed, and the efficiency of catalysis was found to be dependent on the quality of Sm(0) metal used to prepare the SmI_2_ catalyst stock solution; factors that likely affect every catalytic protocol using SmI_2_ (Scheme 9c). The choice of alkene partner is limited to activated electron‐deficient alkenes; use of an electrophilic alkene likely results in efficient intermolecular trapping of the electron‐rich secondary alkyl radical formed upon fragmentation of the BCB and housane ring systems.

While the radical relay approach to catalysis typically requires SmI_2_ alone, leading to high atom economy and operational simplicity, the approach is currently limited to a specific family of substrates containing strained rings, whose fragmentation drives catalysis.

## 4 Electrochemical Strategies

Electrocatalysis provided some of the earliest attempts to harness Sm^II^ reagents in a sustainable fashion by facilitating the in situ generation of active Sm^II^ species from stable and inexpensive Sm^III^ pre‐catalysts.^[^ ^36^ ^]^ The experimental setup typically involved an undivided electrochemical cell, although divided cells have been exploited more recently to prevent the competing oxidation of Sm^II^ at the anode. The choice of cathode material is critical, requiring a sufficiently high overpotential to mitigate against hydrogen evolution through parasitic proton reduction. Examples include nickel foam, stainless steel, and Sm(0) rods.^[^ ^37^ ^]^ Furthermore, for sacrificial electrodes, the material needs to be corrosion resistant and have limited toxicity and low cost.^[^ ^37^ ^]^ The approach involves the electrochemical reduction of a Sm^III^ salt (e.g., Sm(OTf)_3_ or SmCl_3_) at the cathode surface to generate Sm^II^_,_ which diffuses from the electrode into the bulk solution where it acts as a homogeneous chemical reductant before migrating back to the electrode surface as Sm^III^ to close the catalytic cycle. The organic substrate, having accepted an electron to form a radical anion intermediate, subsequently undergoes chemical transformation, such as dimerization or cyclization, to yield the target product. The role of the anode can vary; typically, a sacrificial anode is made of Mg(0) or Al(0), while an inert anode is made of Pt(0) or carbon. In the latter case, the electrolyte is oxidized rather than the anode in order to provide the electrons needed to close the circuit and conserve charge. There are several key considerations when implementing electrocatalytic Sm^II^ processes: i) for sacrificial anode systems, the oxidation potential of the anode must be lower than that of the substrate, product, or solvent, such that it is preferentially oxidized; ii) the choice of solvent greatly differs from those used in the previously discussed strategies for Sm^II^ catalysis; aprotic polar solvents are commonly used as they can maintain homogeneity in the bulk solution while also providing a suitable window of electrochemical stability and conductivity; iii) weak proton donors or alkoxide scavengers are also included in the bulk solution to quench Sm^III^‐bound intermediates. These additives must not be so reactive as to compete for reduction at the cathode surface; finally, iv) the reduction potential of the organic components needs to be more negative than that of the Sm^III^/Sm^II^ redox couple to ensure efficient electron transfer; substrates bearing other reducible functional groups can lead to side reactions that compromise yields.

### 4.1 Early Electrochemical Strategies for Sm^II^ Regeneration

**Scheme 10:**
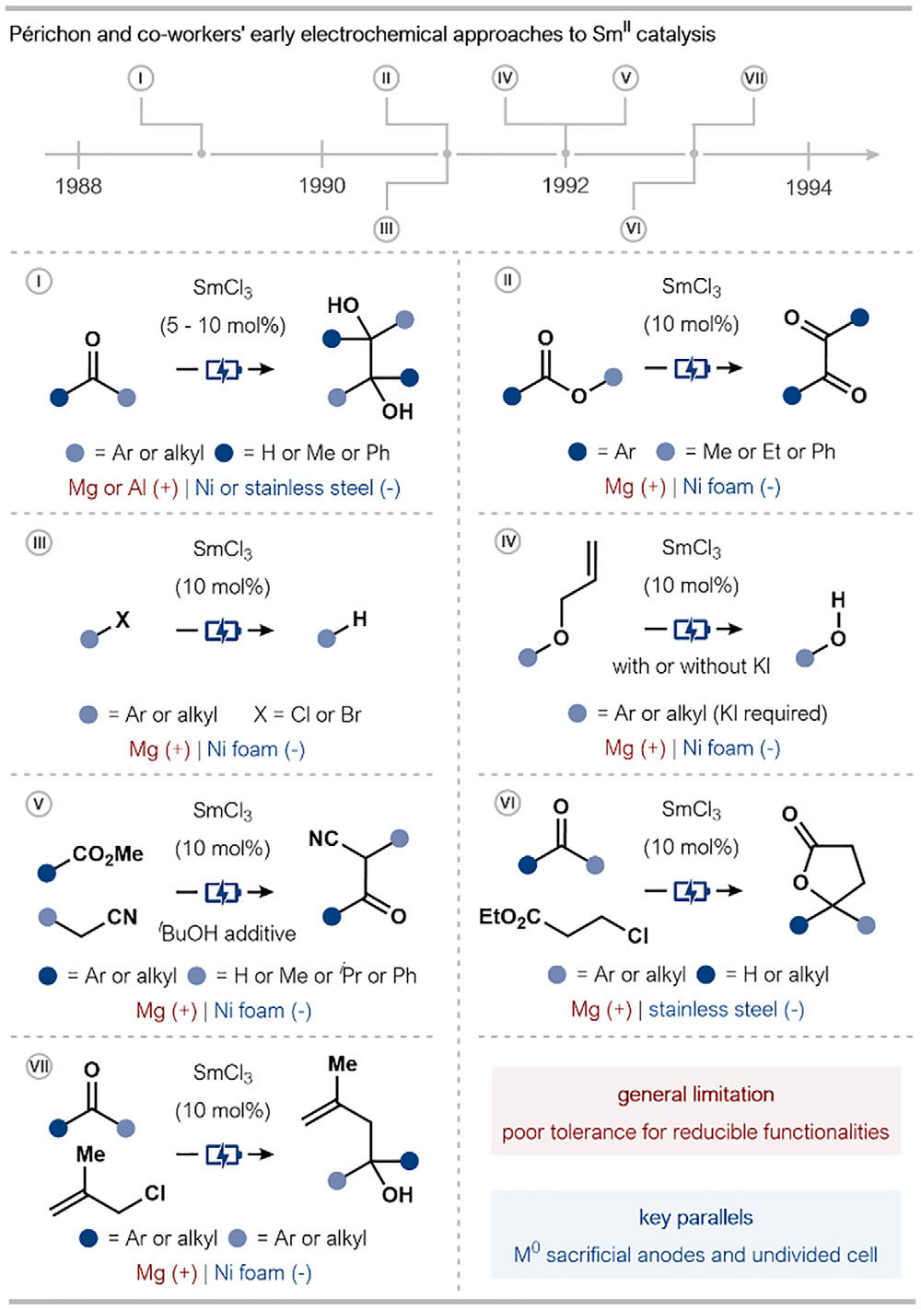
Overview of the early work reported by Périchon and coworkers on electrochemically enabled Sm^II^‐catalysis.

The development of electrochemical strategies for Sm^II^ catalysis in organic synthesis was pioneered by Périchon and coworkers in the late 1980s/early 1990s (Scheme 10).^[^ ^38^ ^]^ Generally, these approaches utilized 10 mol% of the bench‐stable Sm^III^‐precatalyst, SmCl_3_; the active Sm^II^ species was first generated and then continuously regenerated through electrochemical reduction at the cathode. The approach proved highly effective for a range of reductive transformations. A consistent feature across these early reports was the use of a simple, undivided electrochemical cell equipped with a sacrificial metal anode in conjunction with either a stainless steel or nickel foam cathode. Further commonalities include the employment of aprotic solvents with the addition of tetraalkylammonium salts as supporting electrolytes.

In their preliminary work, Périchon and coworkers reported the pinacol coupling of aldehydes and ketones (Scheme 10, Reaction I).^[^ ^38^ ^]^ This was followed by a series of reports, which expanded the scope of the approach to include the selective reductive dimerization of aromatic esters to produce 1,2‐diketones and the efficient reduction of organic halides (Scheme 10, Reactions II and III).^[^ ^39^, ^40^ ^]^ The former is notable for its improved selectivity compared to direct electrochemical reduction in the absence of a catalyst. In 1992, Périchon and coworkers reported two distinct Sm^II^‐catalyzed reactions: the selective cleavage of allyl ethers as a deprotection strategy and the *C*‐acylation of nitriles with esters to give β‐oxonitriles, using *tert*‐butyl alcohol as a pro‐base (Scheme 10, Reactions IV and V).^[^ ^41^, ^42^ ^]^ A further advancement was reported in 1993 with the one‐step electrosynthesis of γ‐butyrolactones from 3‐chloroesters and carbonyl compounds, a method that afforded superior yields and selectivity compared to conventional stoichiometric SmI_2_‐mediated reactions (Scheme 10, Reaction VI).^[^ ^43^ ^]^ In the same year, it was also reported that a similar system could facilitate the allylation of ketones using methylallyl chloride (Scheme 10, Reaction VII).^[^ ^44^ ^]^

Despite the broad utility of their electrochemical approach, certain limitations and nuances were identified. A recurring issue was the competing reduction of functional groups compromising the chemoselectivity of processes. For instance, in the dimerization of aromatic esters, the presence of a halogen substituent (*ortho*‐Cl or *para*‐Br) led to dehalogenation prior to the targeted homocoupling.^[^ ^40^ ^]^ This chemoselectivity issue was also observed in the allyl ether cleavage studies, where aryl halides (*ortho*‐Cl or ‐Br) were reduced before the *O*‐allyl bond was cleaved.^[^ ^41^ ^]^ Interestingly, in the first‐developed pinacol coupling, an aryl *para*‐Cl substituent was tolerated; this improved selectivity may arise from the use of an Al(0) anode in this case, highlighting the subtle relationship between the electrochemical set‐up and selectivity.^[^ ^38^ ^]^ Mechanistic investigations suggested a critical role for the in situ generated Sm^II^ species as both the presence of the catalyst and continuous electrolysis were required to achieve product formation in high yields. Furthermore, in the *C*‐acylation of nitriles, it was proposed that Mg^II^ cations produced at the Mg anode were key, activating the carbonyl group and facilitating the coupling. When an Al(0) anode was employed, no targeted product was obtained.

These early electrochemical strategies by Périchon and coworkers laid the groundwork for the contemporary electrocatalytic Sm^II^ approaches that were to follow.

### 4.2 Sm‐Cathodes for Electrochemical Sm^II^ (Re)Generation

**Scheme 11:**
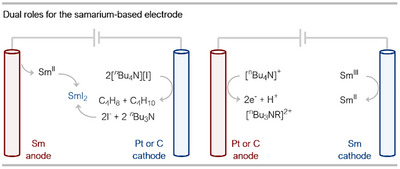
The two roles of the Sm(0) electrode in Sm^II^ electrocatalysis.

More recently, Mellah and coworkers have published a series of reports on the development and application of a versatile electrocatalytic system for Sm^II^‐catalysis. This era of Sm^II^ electrocatalysis is marked by the use of a Sm(0) metal electrode, the function of which is switched during two distinct electrolysis stages (Scheme 11).^[^ ^45^ ^]^ Initially, in a pre‐electrocatalysis step, the Sm rod is employed as a sacrificial anode, during which its electrochemical oxidation generates the active Sm^II^ catalyst in situ when combined with a halide source.^[^ ^46^ ^]^ Following the addition of the substrate, the electrode polarity is reversed, and the Sm(0) electrode subsequently functions as a cathode for the remainder of the reaction, where Sm^III^ is reduced back to Sm^II^ following decomplexation of the product from the Sm center. A common feature in these studies is the employment of an undivided electrochemical cell in conjunction with an oxophilic additive (Me_3_SiCl)—the same additive used in early reports on Sm^II^ catalysis using stoichiometric sacrificial reductants; again, here, the additive cleaves Sm^III^─O bonds, releasing more easily reduced Sm^III^ halide salts for electrochemical regeneration.

**Scheme 12:**
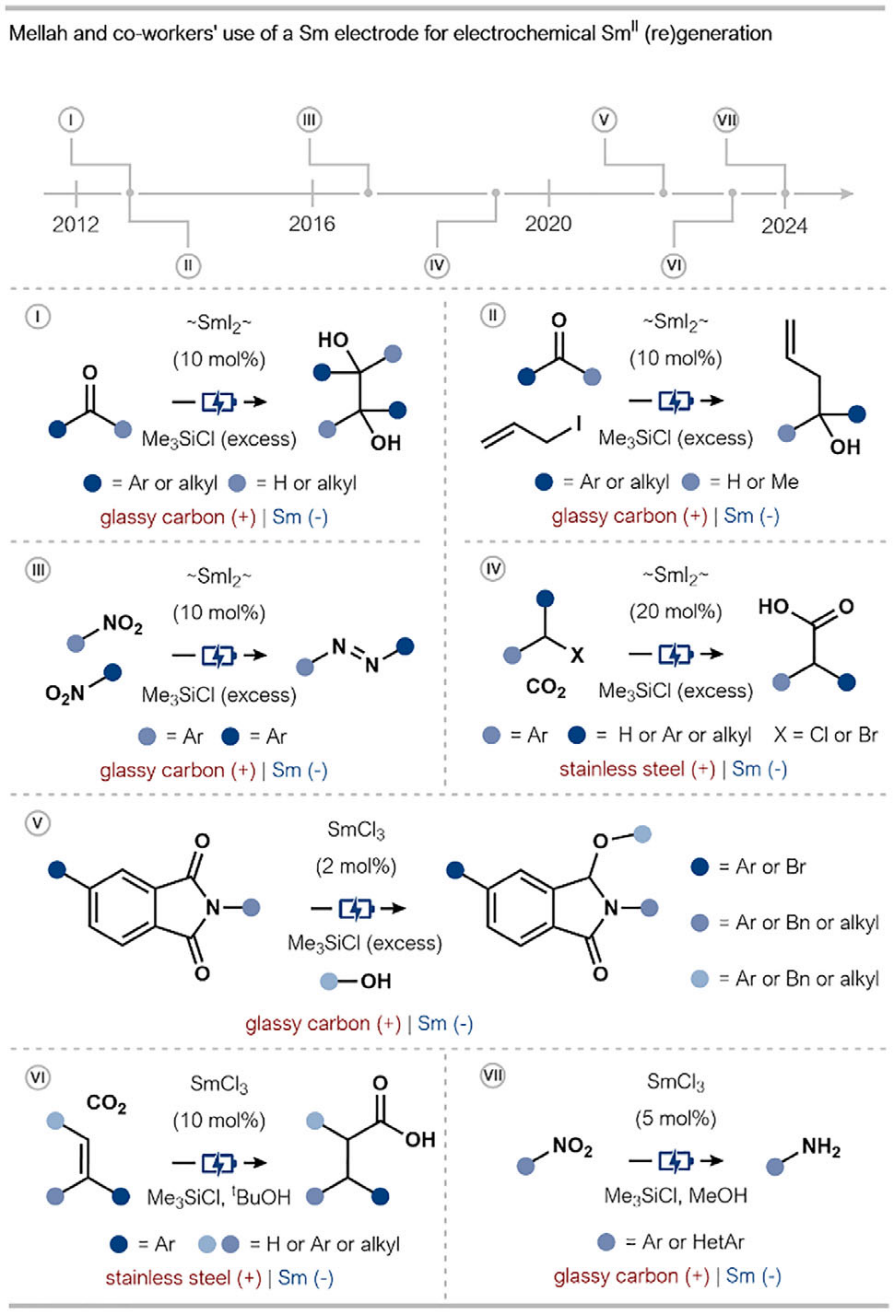
Overview of the work of Mellah and coworkers on the use of a Sm electrode to generate SmI_2_ for Sm^II^‐electrocatalysis. ∼SmI_2_∼: electrochemically generated SmI_2_.

This Sm‐electrode electrocatalytic protocol has been successfully applied to numerous transformations since the seminal report by Mellah and coworkers (Scheme 12). In 2013, the authors reported that electrochemically generated SmI_2_ can be utilized as a catalyst for the pinacol coupling of aryl and alkyl ketones and aldehydes to give 1,2‐diols (Scheme 12, Reaction I).^[^ ^45^ ^]^ In the same report, the authors also disclosed the SmI_2_‐electrocatalyzed Barbier‐type reaction of allyl iodides with aryl and alkyl ketones and aldehydes for the generation of homoallylic alcohols (Scheme 12, Reaction II).^[^ ^45^ ^]^ Subsequently, in 2017, the homo‐ and heterocoupling of nitroarenes to give azobenzenes was reported using similar Sm^II^ electrocatalytic conditions (Scheme 12, Reaction III).^[^ ^47^ ^]^ In 2019, the system was used for the carboxylation of benzyl halides by a CO_2_ activation mechanism; electrogenerated Sm^II^ is thought to reduce CO_2_ (from dry ice) to its radical anion that then undergoes coupling with the benzyl halide partner (Scheme 12, Reaction IV).^[^ ^48^ ^]^ In this study, the choice of solvent and electrolyte had a profound impact on the target product yield, perhaps due to the varying solubility of CO_2_ in different organic solvents. More recently, the Mellah group also reported the Sm^II^‐electrocatalyzed mono‐alkoxylation of *N‐*substituted phthalimides using a range of functionalized alcohols (Scheme 12, Reaction V).^[^ ^49^ ^]^ In this case, SmCl_3_ is used as a precatalyst, reminiscent of the approach of Périchon and coworkers, in conjunction with the use of a Sm electrode.

Building on their interest in CO_2_ activation, in 2023, Mellah and coworkers reported a regioselective anti‐Markovnikov β‐hydrocarboxylation of α‐ or β‐substituted styrenes (Scheme 12, Reaction VI).^[^ ^50^ ^]^ The addition of *^t^* BuOH proved important for obtaining high yields although its role is not clear. Finally, in 2024, the team disclosed how the fate of nitroaromatics under Sm^II^‐electrocatalysis can be switched from the synthesis of azobenzenes to the preparation of anilines simply by swapping the reaction solvent from THF to MeOH (Scheme 12, Reaction VII versus Reaction III).^[^ ^51^ ^]^ This aniline synthesis from nitroarenes is highly chemoselective, tolerating other easily reduced functional groups (e.g., ketones and esters), and proceeds in a flask open to the air. Notwithstanding, a significant excess of Me_3_SiCl is required to achieve reactivity. Again, in these latter two cases, SmCl_3_ is used as a precatalyst in conjunction with the use of a Sm electrode.

The studies of Mellah and coworkers mark a reawakening of interest in the field of Sm^II^‐electrocatalysis with the Sm electrode approach providing a robust and adaptable platform for process development. The systematic studies show how reactions can be directed by the modulation of key parameters, most notably the solvent, the nature and quantity of additives, and the electrolyte anion. For example, the choice between THF and MeOH dictates the outcome of nitroarene reduction, while the presence of an iodide electrolyte is crucial for the efficient carboxylation of benzyl halides. Despite the need for super‐stoichiometric additives and a Sm electrode, the high degree of tunability, combined with the mild conditions and avoidance of toxic co‐reductants, makes the system a significant and versatile tool for contemporary synthesis. While the Sm electrodes are prepared in‐house from Sm rods and stored under an inert atmosphere,^[^ ^45^, ^46^, ^47^, ^48^, ^49^, ^50^, ^51^ ^]^ the authors highlight that the Sm electrode is largely unconsumed during electrolysis and can be reused more than 100 times, although it is not clear if electrode reuse has any effect on catalytic efficiency or product yields.^[^ ^47^ ^]^

### 4.3 Controlled Potential Electrocatalysis

**Scheme 13:**
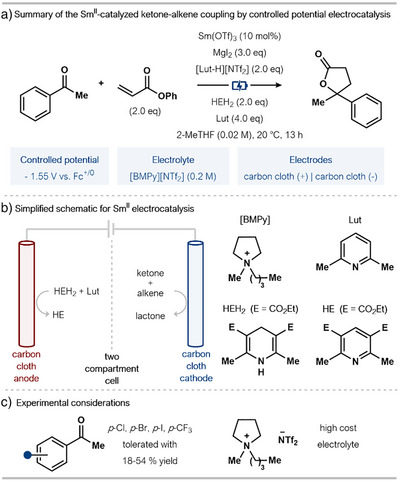
Reisman, Peters, and coworkers Sm^II^‐electrocatalyzed intermolecular coupling of ketones and phenyl acrylate. BMPy: 1‐butyl‐1‐methylpyrrolidinium *bis*(trifluoromethylsulfonyl). Lut: 2,6‐lutidine, 2,6‐dimethyl pyridine.

As part of their 2024 report, Reisman, Peters, and coworkers established proof‐of‐concept with an electrocatalytic manifold for Sm^II^‐catalyzed reductive cross‐couplings (Scheme 13a).^[^ ^23^ ^]^ This system was later applied to electrochemical nitrogen fixation using Mo/Sm co‐catalysis.^[^ ^52^ ^]^ Underpinning their innovative approach is the use of a protonolysis‐based turnover mechanism, wherein LutHNTf_2_ protonates the Sm^III^‐alkoxide intermediate formed after reductive coupling, regenerating a reducible Sm^III^‐halide species (see Section 2.2). A divided electrochemical cell is exploited in conjunction with carbon cloth electrodes, the electrolyte 1‐butyl‐1‐methylpyrrolidinium *bis*(trifluoromethylsulfonyl) imide (BMPyNTf_2_), and an applied potential of −1.55 V (versus Fc^+/0^ (ferrocene)) (Scheme 13b). Additives included MgI_2_ as a halide source, Hantzsch ester (HEH_2_) as a sacrificial reductant at the anode, and an excess of 2,6‐lutidine. 2‐MeTHF was again the optimal solvent (see Section 2.2).

Thorough mechanistic studies, primarily involving extensive cyclic voltammetry studies, demonstrated that the combination of Sm(OTf)_3_, MgI_2_, and LutNTf_2_ generated the key redox‐recyclable SmI_3_ species from an inactive Sm(O*^i^* Pr)_3_ model alkoxide. During reaction optimization, the employment of a sacrificial Zn anode did lead to target product formation (albeit in lower yield) in comparative Faradaic efficiency (FE) while negating the inclusion of HEH_2_ and 2,6‐lutidine. Also, control experiments indicated that the application of a lower potential, −1.65 V versus Fc^+/0^, without the inclusion of a Sm^III^ precatalyst, led to consumption of the starting material but only generated the 1,2‐diol product of homocoupling in 46% yield and with 14% FE.

This result obtained in the absence of Sm highlights the essential role of the Sm mediator in steering the reaction toward the targeted lactone products of cross‐coupling. Of note, reducible C─X bonds are tolerated under the conditions; this represents a significant advance in Sm^II^ electrocatalysis, as, in earlier reports, these functionalities were typically not tolerated (Scheme 13c). The system does, however, require careful potential control to operate below the hydrogen evolution reaction (HER) onset (∼−1.7 V versus Fc^+/0^), is reliant on stoichiometric additives, and employs an expensive electrolyte—although it could be potentially recycled.

As is the case for many carbonyl–alkene couplings, when the alkene is electron‐deficient and reducible, there is some ambiguity around the mechanism of cross‐coupling. The authors refer to the two plausible reduction pathways: “carbonyl‐first” or “alkene‐first.”^[^ ^9^, ^10^, ^53^ ^]^ For aromatic ketones, the data strongly support a “carbonyl‐first” mechanism, as the Sm^II^ re‐oxidation feature in the CV disappears in the presence of the ketone alone, indicating a rapid and irreversible reduction. In contrast, for aliphatic ketones, the initial electron transfer to either the ketone or the acrylate is slow and/or thermodynamically uphill, preventing assignment of the operating mechanism. The role of the Mg^II^ cation was, however, elegantly dissected; CV studies suggest that Mg^II^ facilitates a second electron transfer step to form Mg enolate intermediates.

In summary, by successfully translating their Sm^III^‐alkoxide protonolysis concept to an electrochemical platform, the authors provide a foundation for a new generation of Sm^II^ electrocatalysis.

## 5 Photochemical Strategies

**Scheme 14:**
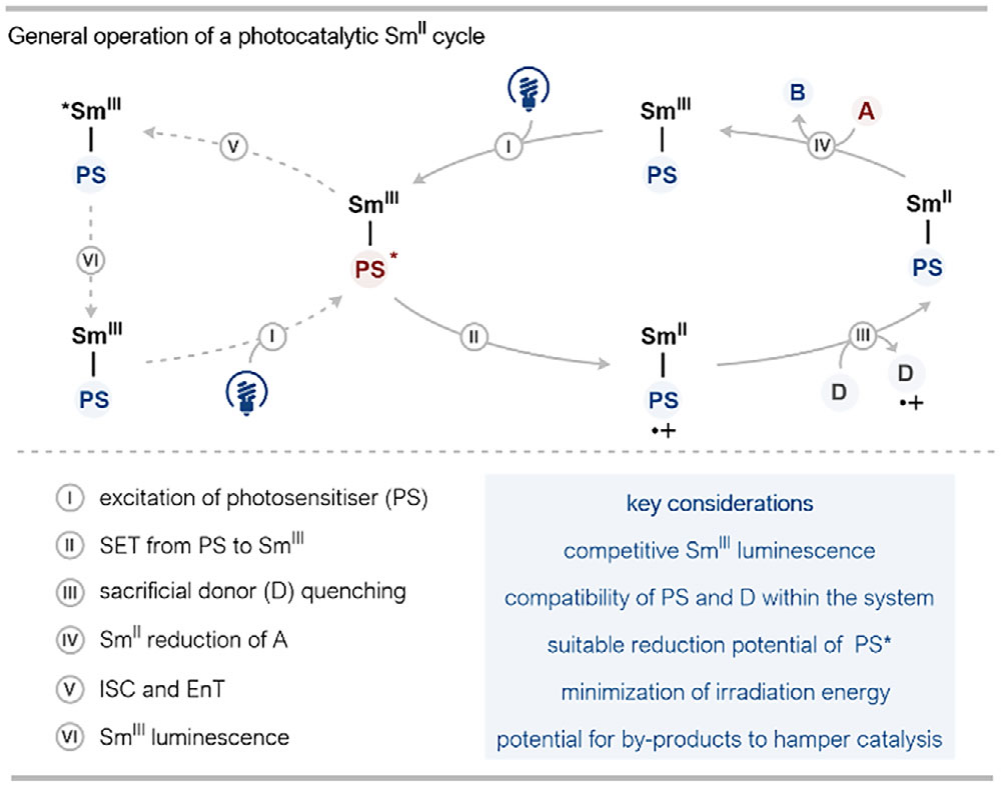
A general photocatalytic cycle involving Sm^II^ bound to a photoactive ligand. A: reducible starting material, B: product of the reaction, D: sacrificial electron donor, EnT: energy transfer, ISC: intersystem crossing, PS: photosensitizer.

Recently, a new approach for Sm^II^ catalysis has emerged that utilizes the energy of visible light to reduce a Sm^III^ salt, armed with a chromophore‐containing ligand, in the presence of sacrificial reductants. A general catalytic cycle can proceed through two distinct pathways when involving a photoactive ligand bound to Sm^II^: an unproductive Sm^III^ luminescence cycle and a productive Sm^II^ catalysis cycle (Scheme 14).^[^ ^54^ ^]^ In the Sm^III^ luminescence cycle (left‐hand cycle), the chromophore (bound to Sm) absorbs light and enters an excited state (Sm^III^–PS*). This photoexcited chromophore then undergoes inter‐system crossing (ISC) and energy transfer (EnT) to the Sm^III^ center generating a Sm^III^ excited state (*Sm^III^) which is quenched through luminescence—this process is intrinsically inefficient owing to the partially forbidden direct 4f→4f electronic transition (Laporte forbidden).^[^ ^55^ ^]^

In the case of the productive Sm^II^ catalytic cycle (right‐hand cycle), the photoexcited chromophore on the Sm^III^‐complex (Sm^III^–PS*) acts as a powerful inner‐sphere reductant and directly reduces Sm^III^ to Sm^II^, generating an Sm^II^–PS^•+^ species. This radical cation can be reductively quenched by an appropriate sacrificial electron donor to form a ground state Sm^II^–PS species, which can carry out the reductive transformation of interest and close the cycle. Key considerations for the successful implementation of this strategy include the quantum efficiency of the photochemical steps, the choice of the photosensitizer, and compatibility of the sacrificial donor in the system.

To mitigate undesirable off‐cycle reactivity, photoactive ligands should be designed to meet two key criteria.^[^ ^56^ ^]^ First, their excited states must be sufficiently reducing to facilitate the conversion of Sm^III^ to Sm^II^. Second, the longest possible wavelength (lowest energy) light should be used to minimize side reactions. The above ensures that the energy input precisely targets the desired redox process while preserving the integrity of sensitive functional groups in substrates.

### 5.1 Coumarin‐Based Ligands for Sm for Photoinduced Electron Transfer (PET)

**Scheme 15:**
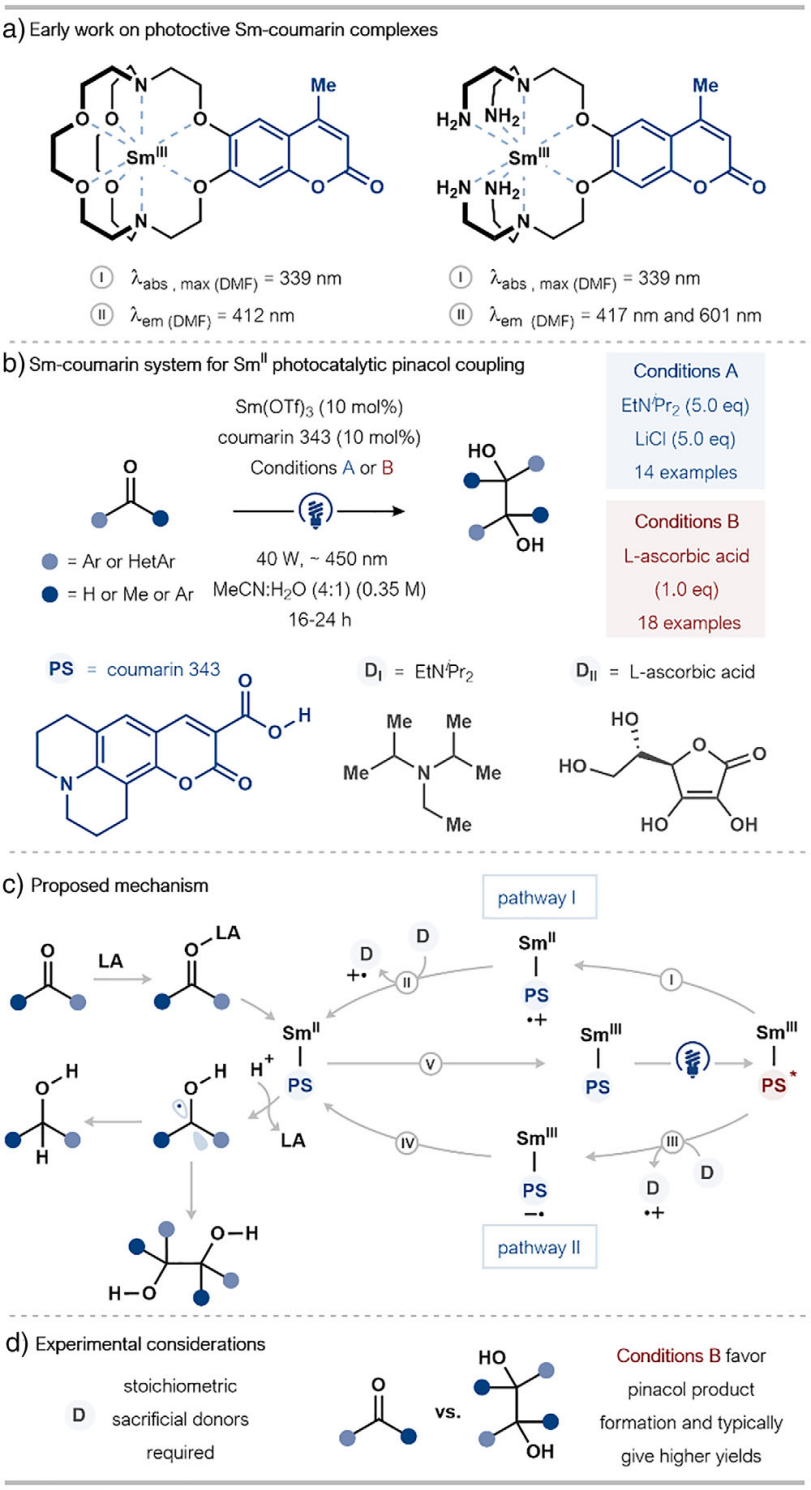
Borbas and coworkers’ photocatalytic Sm^II^ chemistry. a) Early work on the preparation and characterization of Sm^III^–coumarin complexes for photoinduced electron transfer. b) The Sm^III^–coumarin 343 system for Sm^II^‐photocatalyzed pinacol coupling. c) Proposed Sm^II^ photocatalytic cycle. LA: Lewis acid, PS: photosensitizer, D: sacrificial electron donor. d) Highlighted experimental considerations.

In 2023, Borbas and coworkers reported the photocatalytic generation of Ln^II^ reducing agents (Ln = Eu, Sm) by in situ reduction of bench‐stable Ln^III^ precursors bearing photoexcited coumarin ligands on Sm.^[^ ^54^ ^]^ Although the study mainly focused on Eu^II^‐catalyzed reactions, two Sm^III^ complexes were prepared and trialed in a number of reactions (Scheme 15a). Building on this, in 2024, the Borbas group reported a Sm^III^‐coumarin manifold for the Sm^II^ photocatalyzed reduction of carbonyl‐containing compounds to generate the corresponding alcohols or 1,2‐diol products of pinacol homocoupling (Scheme 15b).^[^ ^57^ ^]^ Key to their strategy was the employment of visible light for the photochemical (re)generation of Sm^II^ from a Sm^III^ pre‐catalyst, using sacrificial stoichiometric electron donors, such as *N*,*N*‐diisopropylethylamine (EtN*^i^* Pr_2_) or l‐ascorbic acid. Later, the strategy was also applied to nitrogen fixation and to the reduction of other small molecules (e.g., CO, CO_2_, MHCO_3_).^[^ ^58^, ^59^ ^]^

The proposed mechanism begins with photoexcitation of the postulated Sm^III^–PS pre‐catalyst (PS = coumarin 343) to generate a Sm^III^–PS* species (Scheme 15c). Direct evidence for the formation of a Sm^III^–PS species remains elusive; however, indirect support for the proposal comes from the analogous behavior of Eu(OTf)_3_, whose luminescence lifetime decreases from 0.31 ms to 0.24 ms in the presence of the PS; the shortening of the luminescence lifetime is consistent with the PS binding to Eu^III^. The proposed Sm^II^ catalytic cycle continues with the fast internal quenching of the Sm^III^–PS* excited state (due to the heavy atom effect) to generate Sm^II^–PS^•+^ (pathway I), in a thermodynamically favored process (step I, Δ*G*_eT_ = −0.63 eV). This scenario is supported by an experiment involving the irradiation of a DMF solution of PS and Sm(OTf)_3_ in the presence of the radical trap, *N*‐*tert*‐butyl‐α‐phenylnitrone (PBN); this experiment resulted in the detection (by EPR) of a persistent *N*‐centered radical adduct, consistent with the formation and trapping of PS^•+^, which would otherwise be reductively quenched by the sacrificial donor in step II to form the Sm^II^–PS catalyst (Scheme 15c).

An alternative mechanism is also proposed (pathway II), in which Sm^III^–PS* is reduced by a sacrificial donor to give a Sm^III^–PS^•−^ species (step III) that undergoes internal electron transfer (step IV) to form the key Sm^II^–PS complex. Step III is thought to be thermodynamically favorable for both sacrificial donors employed experimentally, and step IV is energetically downhill (Δ*G*_eT_ = −0.33 eV). In either case, the active Sm^II^–PS catalyst can reduce the carbonyl group of a substrate by proposed inner‐sphere SET from the Sm^II^ center, regenerating the Sm^III^–PS species in step V. Of note, the authors state that pathway II is not operational in the case of Conditions B, as quenching of the PS* was not experimentally observed when l‐ascorbic acid was present. Although two sets of conditions were reported, Conditions B utilize less stoichiometric sacrificial donor, is efficient at lower Sm loadings, and consistently provides higher yields and selectivity for the desired pinacol product. Thus, Conditions B represents the most practical and atom‐economical protocol for the photocatalytic transformation (Scheme 15d).

### 5.2 Anthracene‐Based Ligands for Sm for PET

**Scheme 16:**
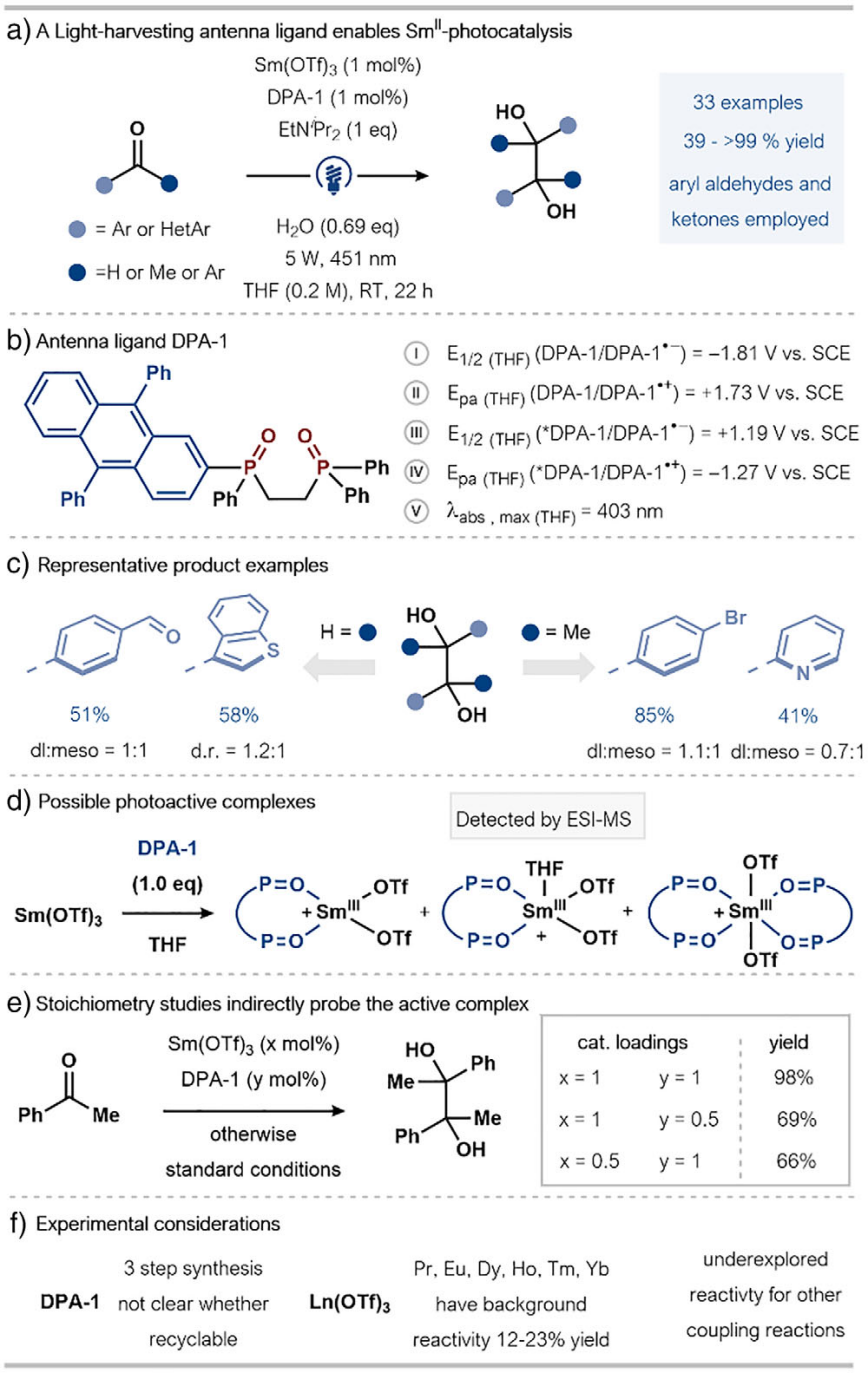
a) Sm^III^–anthracene complexes in a Sm^II^–photocatalytic pinacol coupling of aryl aldehydes and ketones. b) Antenna ligand DPA‐1, DPA: 9,10‐diphenylanthracene. c) Representative scope examples for pinacol coupling. d) Sm–antenna complex structures inferred from ESI‐MS spectroscopic analysis. e) Studies toward the elucidation of the structure of the active Sm‐complex. f) Highlighted experimental considerations.

In 2024, Kuribara, Nemoto, and coworkers reported a visible light‐mediated approach to Sm^II^ catalysis enabled by a bidentate phosphine oxide ligand, DPA‐1, for ligand‐enabled PET (Scheme 16a).^[^ ^60^ ^]^ Akin to the approach of Borbas and coworkers, a Sm^III^ pre‐catalyst is converted to a catalytically competent Sm^II^ species that operates within an overall net reductive catalytic manifold. The DPA‐1 ligand was rationally designed by combining several properties essential for the targeted photocatalytic cycle (Scheme 16b). These included strong coordination affinity arising from the hard phosphine oxide oxygen donor atoms binding effectively to the hard Sm^III^ ion and appropriate photophysical characteristics to function as an efficient visible‐light absorber. Cyclic voltammetry studies, conducted in the reaction solvent, for both DPA‐1 and photoexcited DPA‐1 (*DPA‐1) revealed the ligand has a lower reduction potential than Sm(OTf)_3_ (*E*_pc,red (THF)_ = −1.19 V versus SCE (standard calomel electrode)) and a higher oxidation potential than EtN*^i^* Pr_2_ (*E*_pc,red (THF)_ = +1.11 V versus SCE). Additionally, UV–vis and fluorescence studies determined the absorbance maximum (*λ*_abs_), fluorescence quantum yield (*ϕ*_f _= 0.85), and first singlet excited state energy (*E*(S_1_) = 3.00 eV). This data was used to propose that EtN*^i^* Pr_2_ undergoes reductive SET to *DPA‐1, and the resultant DPA‐1**^•^** ^−^ reduces Sm^III^ to Sm^II^ (pathway II, Scheme 15c). Alternatively, direct internal reductive SET from *DPA‐1 to the Sm^III^ center may occur.

The synthetic utility of the method was demonstrated through its application in the pinacol coupling of aryl aldehydes and ketones featuring other reducible functional groups (e.g., C═O, C─Br bonds) or heteroaromatic ketones that possess additional coordinating moieties (Scheme 16c). Of note, while the yields generally ranged from moderate to good across the reported scope, diastereoselectivity was low.

Additional mechanistic investigations were conducted to elucidate the nature of the active Sm‐DPA‐1 species. To this end, ESI‐MS spectroscopic analysis of a 1:1 mixture of Sm(OTf)_3_ and DPA‐1 revealed the presence of three species: Sm(OTf)_2_(DPA‐1), its THF adduct, and a bis‐coordinated Sm(OTf)_2_(DPA‐1)_2_ species (Scheme 16d). Varying the ratio of Sm to DPA‐1, away from the optimal 1:1 value, resulted in decreased yield of the diol product in the pinacol coupling of acetophenone; this supports the monocoordinated Sm^II^ species being the active catalyst (Scheme 16e). The interplay between Sm(OTf)_3_, DPA‐1, and EtN*^i^* Pr_2_ was investigated using emission spectroscopy: while EtN*^i^* Pr_2_ can quench *DPA‐1, a much stronger quenching was observed in the presence of Sm(OTf)_3_. Based on these findings, the authors suggest a dynamic equilibrium between coordinated and free Sm‐complexes that influences photophysical behavior and subsequent reactivity.

In summary, this work presents an alternative approach to visible‐light‐driven Sm^II^‐catalysis, as exemplified in pinacol‐type couplings using an anthracene‐based ligand for Sm. While the optimal ligand requires a multistep synthesis, recycling may be possible. A modest background reaction to give pinacol product is seen when alternative, presumably redox‐inactive, Ln^III^ salts were employed in lieu of Sm(OTf)_3_. This underlines that Sm is not simply acting as a Lewis acid in activating substrate to a background, direct reduction by *DPA‐1 (Scheme 16f). Crucially, the Sm^II^ photocatalytic system was applied to other transformations typically mediated by SmI_2_—e.g., ketone–alkene coupling and keto‐epoxide opening—with promising preliminary results. Thus, the approach shows significant potential for wider future application.

### 5.3 Approaches Using External Photocatalysts

**Scheme 17:**
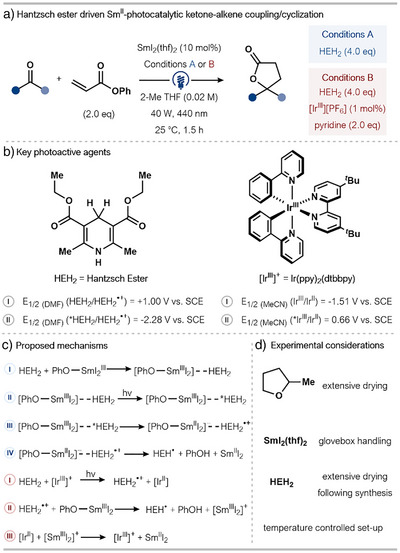
a) Photocatalytic Sm^II^‐catalyzed ketone–alkene coupling/cyclization sequence using a Hantzsch ester as a stoichiometric reductant. b) Key photoactive agents with their corresponding redox potentials. c) Proposed mechanism: blue I–IV refers to Condition A and red I–III refers to Condition B. d) Highlighted experimental considerations. dtbbpy: 4,4‐di‐*tert*‐butyl‐2,2‐dipyridyl, ppy: 2‐phenylpyridyl.

In 2024, Peters and coworkers reported a photocatalytic approach to catalysis with Sm^II^, in which an external photocatalyst is used to regenerate Sm^II^ from Sm^III^ and Hantzsch ester (HEH_2_) is used as the terminal reductant. This proof‐of‐concept study was applied to the Sm^II^‐catalyzed intermolecular ketone–alkene cross‐coupling and lactonization seen earlier (see Section 2.2) (Scheme 17a).^[^ ^61^ ^]^

Guided by literature precedent, it was proposed that photoexcited HEH_2_ (*HEH_2_) would be sufficiently reducing to generate Sm^II^ from Sm^III^ (Scheme 17b).^[^ ^62^ ^]^ Nonetheless, the authors highlighted that HEH_2_ is reported to have a low quantum yield and short excited state lifetime, which is likely responsible for the modest yields of Sm^II^ obtained in initial stoichiometric Sm^III^‐to‐Sm^II^ studies. A protocol using the Ir^III^‐photoredox catalyst, [Ir(dtbbpy)(ppy)_2_][PF_6_], capable of generating a highly reducing Ir^II^‐species and converting Sm^III^ to Sm^II^ using a sacrificial electron donor, was therefore developed (Scheme 17b).^[^ ^63^, ^64^, ^65^ ^]^

Using HEH_2_ as a direct photo‐reductant, the mechanism begins with pre‐association of HEH_2_ to a Sm^III^‐alkoxide, supported by an observed bathochromic shift in the UV–vis absorbance of HEH_2_ in the presence of model SmI_2_(O*^i^* Pr) (Scheme 17c). As the successful coupling/lactonization involves phenyl acrylate as the alkene partner, Sm^III^‐phenoxide [PhO–Sm^III^I_2_] is formed and is proposed to pre‐associate with HEH_2_. Next, excitation of the ligated HEH_2_ generates a Sm^III^ complex [PhO–Sm^III^I_2_]‐*HEH_2_, which undergoes both an electron transfer—forming intermediate [PhO–Sm^II^I_2_]‐HEH_2_^•+^—and proton transfer to access the key Sm^II^ species, free PhOH, and HEH^•^. In the case of the Ir^III^‐photoredox system, photoexcited *[Ir^III^]^+^ is reductively quenched by sacrificial HEH_2_ to generate [Ir^II^] and HEH_2_^•+^. HEH_2_^•+^ then undergoes proton transfer to a Sm^III^‐phenoxide to liberate a cationic [Sm^III^I_2_]^+^ that can be reduced to Sm^II^ by [Ir^II^] (Scheme 17c).

This study represents an important departure from previous photochemical strategies for Sm^II^ catalysis, amalgamating the established field of photocatalysis with Sm^II^ chemistry, without the need to prepare a photoactive Sm^II^ complex. However, the use of SmI_2_(thf)_2_, an air‐ and moisture‐sensitive solid, as the Sm^II^ source does necessitate rigorous exclusion of O_2_ and H_2_O (Scheme 17d). This sensitivity imposes operational constraints; a glovebox set up and carefully dried solvents are required to maintain reagent integrity. Perhaps the most striking feature of the approach is the use of a mainstream, commercial Ir^III^ photocatalyst, for the first time, in a proposed dual‐catalytic system, to address the challenge of catalysis with SmI_2_.

## 6 Future Outlook

This review provides a detailed summary of the various approaches taken to achieve catalysis with SmI_2_. Given the interest in the unique reactivity and selectivity of SmI_2_ chemistry over the past four decades, surprisingly few studies dedicated to developing catalytic Sm^II^ systems have appeared, a testament to the many challenges involved. That said, recent years have seen significant progress made, using stoichiometric metal reductants in conjunction with catalytic SmI_2_ alongside the emergence of innovative Sm^II^ catalytic systems that borrow, for example, from contemporary photo‐ and electrocatalysis. Several of these approaches also involve a switch to using user‐friendly Sm^III^ precatalysts, with Sm^II^ generated in situ. Furthermore, so‐called radical‐relay, redox‐neutral approaches using SmI_2_ alone have been developed that provide valuable products and new insights into the reversibility of Sm^II^ reductions and the properties of Sm‐bound radicals.

While new approaches to Sm^II^ catalysis still suffer from limitations and are far from general in terms of the types of transformations they mediate and the selectivity that can be achieved, every step forward provides crucial mechanistic understanding, advancements in analytical techniques and computational modeling, and the promise of a new chapter in the chemistry of SmI_2_. In particular, recent photocatalytic systems stand out for their use of rationally designed ligand classes that harvest visible light to drive Sm^II^ catalysis. The design of more effective ligand systems that better control the coordination chemistry and stability of Sm complexes promises to unlock more efficient catalysis at lower loadings of Sm.

In summary, rather than a “one‐size‐fits‐all” approach to catalysis with SmI_2_, perhaps a suite of practical systems for Sm^II^ catalysis will soon provide the generality needed for widespread take‐up by the synthetic community. As the technologies come together, and catalytic capability takes shape, asymmetric redox catalysis with SmI_2_, using chiral ligands on Sm,^[^ ^66^ ^]^ will also likely become possible. Ultimately, the ability to exploit the uniquely rich and selective chemistry of SmI_2_ in catalysis promises to introduce the reagent to a new generation of scientists, dedicated to more sustainable synthesis, working across many disciplines in academia and industry, on various scales and using a range of reaction platforms, helping them solve synthetic problems and efficiently deliver molecules of societal importance.

## Conflict of Interests

The authors declare no conflict of interest.

## Data Availability

Data sharing is not applicable to this article as no new data were created or analyzed in this study.
